# Modeling of the Time-Dependent H_2_ Emission and Equilibrium Time in H_2_-Enriched Polymers with Cylindrical, Spherical and Sheet Shapes and Comparisons with Experimental Investigations

**DOI:** 10.3390/polym16152158

**Published:** 2024-07-29

**Authors:** Jae Kap Jung, Ji Hun Lee, Jae Yeong Park, Sang Koo Jeon

**Affiliations:** 1 Hydrogen Energy Group, Strategic Technology Research Institute, Korea Research Institute of Standards and Science, Daejeon 34113, Republic of Korea; jkjung@kriss.re.kr (J.K.J.); ljh93@kriss.re.kr (J.H.L.); sangku39@kriss.re.kr (S.K.J.); 2 Department of Measurement Science, University of Science and Technology, 217 Gajeong-ro, Yuseong-gu, Daejeon 34113, Republic of Korea

**Keywords:** enriched polymers, numerical modeling, hydrogen desorption equilibrium time, hydrogen uptake, diffusion, diffusion analysis program

## Abstract

Time-dependent emitted H_2_ content modeling via a reliable diffusion analysis program was performed for H_2_-enriched polymers under high pressure. Here, the emitted hydrogen concentration versus elapsed time was obtained at different diffusivities and volume dimensions for cylinder-, sphere- and sheet-shaped specimens. The desorption equilibrium time, defined as the time when the H_2_ emission content is nearly saturated, was an essential factor for determining the periodic cyclic testing and high-pressure H_2_ exposure effect. The equilibrium time in the desorption process was modeled. The equilibrium time revealed an exponential growth behavior with respect to the squared thickness and the squared diameter of the cylinder--shaped specimen, while it was proportional to the squared diameter for the sphere-shaped specimen and to the squared thickness for the sheet-shaped specimen. Linear relationships between the reciprocal equilibrium time and diffusivity were found for all shaped polymers. The modeling results were confirmed by analysis of the solutions using Fick’s second diffusion law and were consistent with the experimental investigations. Numerical modeling provides a useful tool for predicting the time-dependent emitted H_2_ behavior and desorption equilibrium time. With a known diffusivity, a complicated time-dependent emitted H_2_ behavior with a multi-exponential form of an infinite series could also be predicted for the three shaped samples using a diffusion analysis program.

## 1. Introduction

Gas sorption is a main physical process in polymer media, and desorption is the reversal process controlled by Fick’s diffusion laws. H_2_ molecule sorption and desorption are the predominant parameters used to determine the solubility, diffusivity and permeation of gas-sealed polymer in hydrogen infrastructure [1,2,3,4,5,6]. Moreover, the fracture phenomenon caused by H_2_ embrittlement in O-ring seals is related to the residence time of the penetrated H_2_ molecules in the polymer network during the sorption and desorption processes. Additionally, the H_2_ transport characteristics are associated with the equilibrium features related to the diffusion mechanism of both processes under high-pressure environments [7,8].

The H_2_ equilibrium time is described as the saturated attainment of nearly the maximum H_2_ content in a polymer through the sorption and desorption processes and is affected predominantly by the sample shape, dimension and diffusion coefficient. Investigations of the saturated equilibrium time after high-pressure exposure in hydrogen permeation are essential for designing durable O-rings, reducing operating costs, gaining insights into adsorption and, finally, determining the appropriate exposure time of H_2_ under high pressure through the cycling tests [9,10].

On the other hand, the H_2_ diffusivity and desorbed (absorbed) content in gas-enriched polymers are mainly dependent on the filler spices and content [11,12,13,14,15]. For the measurement of the permeation parameters after hydrogen exposure in the polymers with different fillers, the same exposure conditions are needed. The same exposure conditions imply the equilibrium time in sorption and desorption processes. Specifically, the measurements of the hydrogen exposure effect need to be performed after the H_2_ sorption equilibrium time is reached. Thus, we should determine the equilibrium time of the hydrogen sorption and desorption before starting the measurement. In a previous investigation, the equilibrium times of sorption and desorption were found to be the same, with a margin of nearly 10%. The polymer specimens normally have different H_2_ diffusivity ranging from 10^−9^ m^2^/s to 10^−11^ m^2^/s.

In this study, we modeled only the time dependence of the emitted H_2_ content and the equilibrium time in the desorption process for the cylinder-, sphere- and sheet-shaped polymer samples with different thicknesses, diameters and diffusivities of corresponding ranges without measurements. The desorption equilibrium time is important for determining the high-pressure exposure conditions in the periodic cycling test of rubbers and designing O-ring materials for high-pressure hydrogen gas sealing devices.

The time-dependent behavior of the emitted H_2_ content and its equilibrium time were evaluated by performing numerical modeling based on the solutions from Fick’s second law; this was conducted by employing a validated diffusion analysis program [16,17,18,19]. In particular, the relationships of the equilibrium time with respect to the specimen shape, dimension and diffusivity are investigated for the three shaped specimens, which are well consistent with the experimental results. Consequently, this research provides a useful tool for determining the equilibrium time of H_2_ and the time evolution of desorption patterns after specimens are exposed to high pressure with different diffusivities. For the case of a known diffusivity, a complicated time-dependent emitted H_2_ behavior with a multi-exponential form of an infinite series can be predicted for shaped samples with any dimension by employing a diffusion analysis program.

## 2. Modeling Background

### Modeling Background for Determining the Sorption Equilibrium Time

In the presence of gas concentration gradients, H_2_ is absorbed into a rubber specimen and diffuses across the specimen to permeate to the opposite side. The diffusion flux (*J*) is the flow of the specimen through a unit cross-sectional area in a unit time and is defined as Equation (1) [20,21,22].

(1) $$J=-D\frac{dC}{dx}$$

where D is the H_2_ diffusivity and C is the H_2_ concentration. Fick’s first law describing steady-state diffusion relates the diffusion flux to the change in the H_2_ concentration.

The dissolution of H_2_ into a specimen is described by Henry’s law, c=Sp [23], where S is the solubility and *p* is the pressure. Thus, Equation (1) can be expressed as follows:

(2) $$J=-DS\frac{dp}{dx}=-P\frac{dp}{dx}$$

where P is the permeability expressed as P=DS.

Under unsteady conditions, the concentration of diffusing H_2_ changes over time and is described by Fick’s second law of diffusion as follows [21]:

(3) $$\frac{\partial C}{\partial t}=\frac{\partial }{\partial x}(D\frac{\partial C}{\partial x})$$

Assuming that the H_2_ desorption is governed by a diffusion process, the emitted H_2_ concentration $C_{E}(t)$ in the desorption process is expressed as Equation (4) [24,25]:

(4) $$\frac{C_{E}(t)}{C_{\infty }}=1-\frac{32}{\pi^{2}}\times \left[\sum_{n=0}^{\infty }\frac{exp\left\{\frac{{-\left(2n+1\right)}^{2}\pi^{2}Dt}{l^{2}}\right\}}{{\left(2n+1\right)}^{2}}\right]\times \left[\sum_{n=1}^{\infty }\frac{exp\left\{-\frac{D\beta_{n}^{2}t}{\rho^{2}}\right\}}{\beta_{n}^{2}}\right]$$

Equation (4) shows the solutions to Fick’s second diffusion law for a cylinder-shaped specimen, where the boundary condition for a constantly uniform H_2_ concentration is initially maintained and the cylindrical surfaces are kept at a constant concentration. In Equation (4), l and ρ are the thickness and radius, respectively, of the cylindrical rubber sample, and $\beta_{n}$ is the root of the zero-order Bessel function. In Equation (4), $C_{\infty }$ is the saturated emitted hydrogen mass concentration at an infinitely long time, i.e., hydrogen uptake in the desorption process. D is the desorption diffusion coefficient.

The equilibrium time (*t_eq_*) in the desorption process can be defined as the time at which the emitted H_2_ content reaches 97% of the total sorption content, i.e., *C_E_*(*t = t_eq_*) = 0.97 $C_{\infty }$. The dependence of the equilibrium time on the sample shape and diffusivity was investigated for cylinder-, sphere- and sheet-shaped specimens.

For cylindrical specimens with a fixed thickness ($l_{fixed}$), a fixed diameter ($\rho_{fixed}$) and $\frac{C_{E}(t_{eq})}{C_{\infty }}$ = 0.97 at equilibrium, Equation (4) can be written as follows:

(5) $$\frac{32}{\pi^{2}}\times \left[\sum_{n=0}^{\infty }\frac{exp\left\{\frac{{-\left(2n+1\right)}^{2}\pi^{2}Dt_{eq}}{l_{fixed}^{2}}\right\}}{{\left(2n+1\right)}^{2}}\right]\times \left[\sum_{n=1}^{\infty }\frac{exp\left\{-\frac{D\beta_{n}^{2}t_{eq}}{\rho_{fixed}^{2}}\right\}}{\beta_{n}^{2}}\right]=0.03=constant$$

The dependence of the equilibrium time on the diffusivity is as follows: $D\cdot t_{eq}$ = constant. This relationship indicates that $\frac{1}{D}$ is linear with respect to $t_{eq}$.

For the cylindrical specimen with a fixed diffusivity ($D_{fixed}$), a fixed diameter ($\rho_{fixed}$) and $\frac{C_{E}(t_{eq})}{C_{\infty }}$ = 0.97, Equation (4) can be modified as follows:

(6) $$\frac{32}{\pi^{2}}\times \left[\sum_{n=0}^{\infty }\frac{exp\left\{\frac{{-\left(2n+1\right)}^{2}\pi^{2}D_{fixed}t_{eq}}{l^{2}}\right\}}{{\left(2n+1\right)}^{2}}\right]\times \left[\sum_{n=1}^{\infty }\frac{exp\left\{-\frac{{D_{fixed}\beta }_{n}^{2}t_{eq}}{\rho_{fixed}^{2}}\right\}}{\beta_{n}^{2}}\right]=0.03=constant$$

The relationship between the equilibrium time and the squared thickness is not linear but exponential because of the additive second exponential term in Equation (6). Similarly, for the cylindrical specimen, a fixed diffusivity ($D_{fixed}$), a fixed thickness ($l_{fixed}$) and $\frac{C_{E}(t_{eq})}{C_{\infty }}$ = 0.97, Equation (4) can be modified as follows:

(7) $$\frac{32}{\pi^{2}}\times \left[\sum_{n=0}^{\infty }\frac{exp\left\{\frac{{-\left(2n+1\right)}^{2}\pi^{2}D_{fixed}t_{eq}}{l_{fixed}^{2}}\right\}}{{\left(2n+1\right)}^{2}}\right]\times \left[\sum_{n=1}^{\infty }\frac{exp\left\{-\frac{D_{fixed}\beta_{n}^{2}t_{eq}}{\rho^{2}}\right\}}{\beta_{n}^{2}}\right]=0.03=constant$$

The relationship between the equilibrium time and the squared diameter is not linear because of the additive first exponential term in Equation (7).

The emitted H_2_ concentration $C_{E}(t)$ in the desorption process for a spherical sample is expressed as follows [24,26]:

(8) $$\frac{C_{E}(t)}{C_{\infty }}=[1-\frac{6}{\pi^{2}}\sum_{n=1}^{\infty }\frac{1}{n^{2}}exp\left(-\frac{Dn^{2}\pi^{2}t}{a^{2}}\right)]$$

Equation (8) shows the solutions to Fick’s second diffusion law with an initially constant uniform hydrogen concentration and a constant concentration at the spherical surface. $C_{\infty }$ is the hydrogen uptake measured in emission mode. a is the radius of the spherical rubber.

For the sphere-shaped specimen with a fixed diameter ($a_{fixed}$) and $\frac{C_{E}(t_{eq})}{C_{\infty }}$ = 0.97 at equilibrium, Equation (8) can be modified as follows:

(9) $$\frac{6}{\pi^{2}}\sum_{n=1}^{\infty }\frac{1}{n^{2}}exp\left(-\frac{Dn^{2}\pi^{2}t_{eq}}{a_{fixed}}\right)=0.03=constant$$

Thus, $D\cdot t_{eq}$ = constant. This relationship indicates that $\frac{1}{D}$ is linear with respect to $t_{eq}$. Moreover, for the spherical specimen with a fixed diffusivity and $\frac{C_{E}(t_{eq})}{C_{\infty }}$ = 0.97, then $\frac{t_{eq}}{a^{2}}$ = constant. This relationship indicates that $t_{eq}$ is linear with respect to $a^{2}$.

Equation (10) shows the solutions to Fick’s second diffusion law for a plane sheet specimen for the emitted H_2_ concentration $C_{E}(t)$ in the desorption process [24].

(10) $$\frac{C_{E}(t)}{C_{\infty }}=[1-\frac{8}{\pi^{2}}\sum_{n=0}^{\infty }\frac{1}{{\left(2n+1\right)}^{2}}exp\left(-\frac{D{\left(2n+1\right)}^{2}\pi^{2}t}{l^{2}}\right)]$$

where *l* is the thickness of the sheet-shaped rubber. Similarly, for a fixed-thickness sheet-shaped specimen and $\frac{C_{E}(t_{eq})}{C_{\infty }}$ = 0.97, then $D\cdot t_{eq}$ = constant. This relationship indicates that $\frac{1}{D}$ is linear with respect to $t_{eq}$. Moreover, for the sheet-shaped specimen with a fixed diffusivity and $\frac{C_{E}(t_{eq})}{C_{\infty }}$ = 0.97, then $\frac{t_{eq}}{l^{2}}$ = constant. This relationship indicates that $t_{eq}$ is linear with respect to $l^{2}$.

## 3. Modeling for Three Shaped Specimens and Comparison with Experiment

To produce the time-varying H_2_ concentration curve of multi-exponential functions with infinite series in Equations (4), (8) and (10), we developed a dedicated diffusion analysis program using Visual Studio 2019 written in c# language, based on a least-squares regression and the Nelder–Mead simplex optimization algorithm [18,27]. The program calculation included the summation of all the contributions up to *n* = 50–100 terms in infinite series.

Equations (4), (8) and (10) are dependent on the dimension and diffusivity of the specimen. Due to the modeling process of the analysis program, the H_2_ emission behavior for the different diffusivities with different cylindrical thicknesses and radii, different spherical radii and different thicknesses of the sheet-shaped specimen, together with the desorption equilibrium times, can be predicted. The equilibrium times modeled for the remaining and emitted contents are the same. Thus, we modeled the desorption equilibrium time in emission mode with a validated diffusion analysis program [16,17,18,19]. The algorithm and code for the analysis program are described in Appendix A with an application example of the diffusion analysis program to obtain the diffusion coefficient and hydrogen uptake amount for NBR exposed to 8.9 MPa. In addition, the modeling results are compared with experimental results.

**Figure 1:**
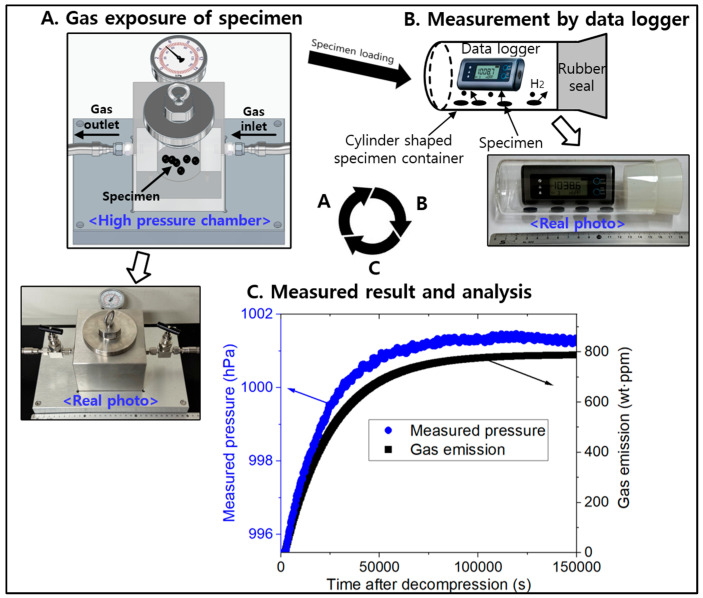
Diagram of manometric measurement sequence to measure the concentration and diffusivity of the emitted H_2_. (**A**) High-pressure chamber for H_2_ exposure. (**B**) Cylinder-shaped specimen container with a USB-type data logger. (**C**) Results for measured pressure versus time together with analysis.

The measurement methods and principle are found in the previous literatures [16,17,18]. Figure 1 shows the diagram of manometric measurement sequence to measure the concentration and diffusivity of the emitted H_2_. The system consists of high-pressure chamber for H_2_ exposure in Figure 1A, cylinder-shaped specimen container with a USB-type data logger in Figure 1B. The commercial sensors used for the pressure/temperature measurements are data loggers to measure and record both the atmospheric pressure and temperature. Figure 1C is the results for measured pressure versus time together with analysis.

After the exposure and the decompression from the chamber in Figure 1A, the specimen was loaded in the cylindrical container in Figure 1B. The H_2_ released from the specimen increased the pressure in the specimen container with increasing time. Thus, the pressure (P) and temperature (T) of the gas inside the sample container varied with time. The gas in container was governed by ideal gas equation: *PV* = *nRT*, where R is the gas constant and *n* is mole number of emitted H_2_ in the specimen container. The principle and measuring processes for obtaining H_2_ concentration and diffusivity, including volumetric method, are detail described in the literature [16,18].

For experimental investigations, we employed the polymer specimens, such as nitrile butadiene rubber (NBR), ethylene propylene diene monomer (EPDM) and fluoroelastomer (FKM), which are used as sealing materials in the hydrogen refueling station. A low-density polyethylene (LDPE) sample was contained, which is employed as a plastic pipeline for H_2_ transport. The polymer specimens are actually used as gas-sealing materials in the hydrogen infrastructure. Therefore, the investigations of the saturated equilibrium time and the permeation properties in H_2_ infrastructure are essential for determining the appropriate exposure time/permeation of H_2_ for the cycling test. Thus, we selected the polymers NBR, EPDM, FKM and LDPE, which are actually used in H_2_ environments. In addition, the basic properties, application fields, chemical compositions and density of polymers are already described in the literatures [17].

### 3.1. Modeling for the Cylinder-Shaped Polymer Specimens and Comparison with Experimental Results

**Figure 2:**
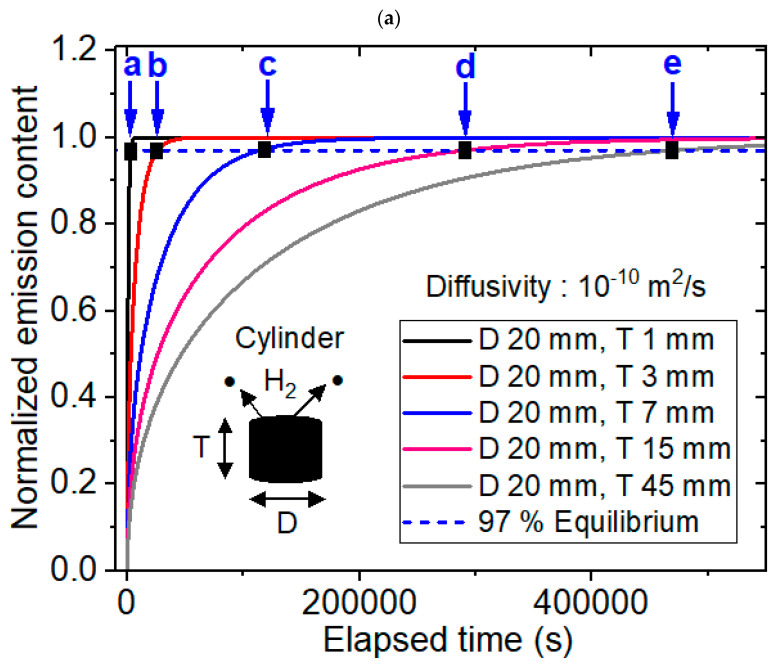

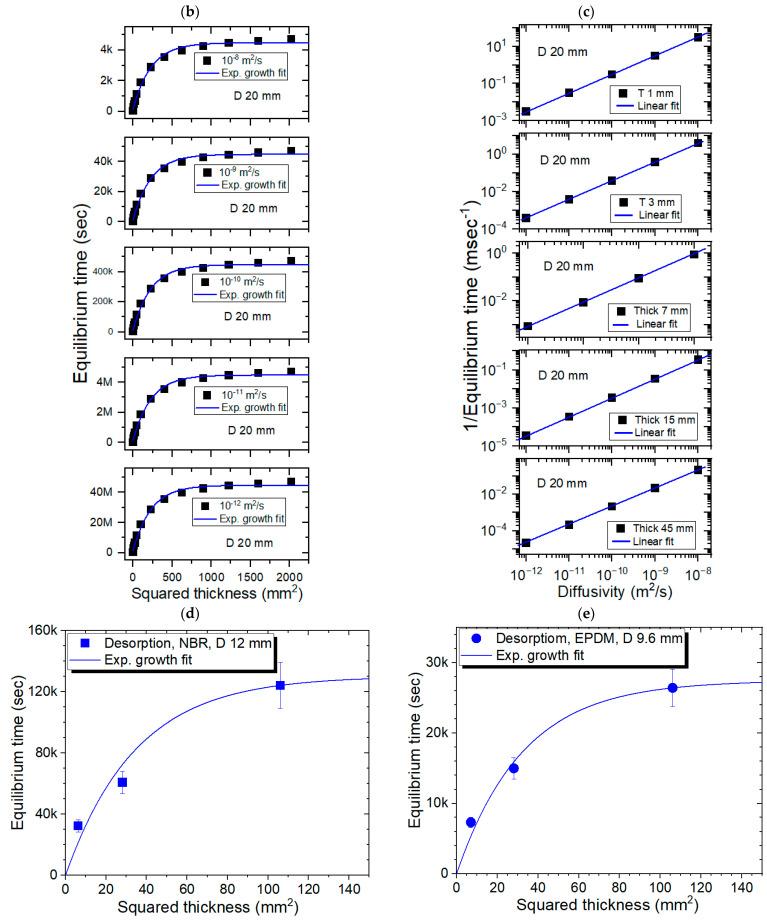

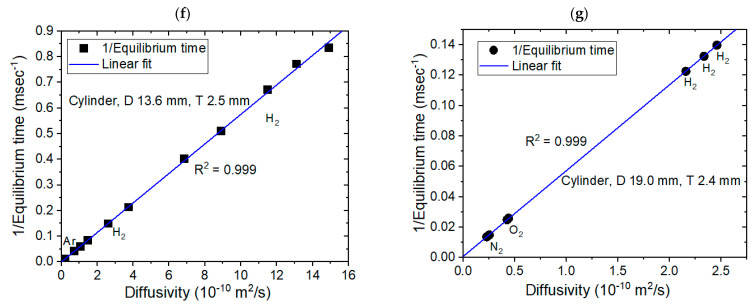
Modeling (**a**–**c**) and experimental (**d**–**g**) results for the cylinder-shaped specimen with a fixed diameter. (**a**) Numerical modeling result for the normalized hydrogen desorption content versus the elapsed time for a diffusivity of 1 × 10^−10^ m^2^/s and five different thicknesses at a fixed diameter of 20 mm. (**b**) Numerical modeling result for the hydrogen equilibrium time versus the squared thickness with five different diffusivities at a fixed diameter of 20 mm. (**c**) Linear correlation between the reciprocal equilibrium time and diffusivity with five different thicknesses at a diameter of 20 mm. (**d**) Experimental results with the desorption equilibrium time versus the squared thickness at a fixed diameter of 12 mm in the NBR specimen. (**e**) Experimental results with the desorption equilibrium time versus the squared thickness at a fixed diameter of 9.6 mm in the EPDM specimen. (**f**) Experimental results with reciprocal equilibrium time versus the diffusivity at a fixed diameter of 13.6 mm and a fixed thickness of 2.5 mm. (**g**) Experimental results with reciprocal equilibrium time versus the diffusivity at a fixed diameter of 19.0 mm and a fixed thickness of 2.4 mm. D and T in Figure 2 indicate the diameter and thickness, respectively, of the cylindrical specimen.

Figure 2 shows modeling and experimental results for the cylinder-shaped specimens. Modeling of cylinder-shaped specimens was performed using a dedicated diffusion analysis program with five different diffusivities of 1 × 10^−8^ m^2^/s, 1 × 10^−9^ m^2^/s, 1 × 10^−10^ m^2^/s, 1 × 10^−11^ m^2^/s and 1 × 10^−12^ m^2^/s and thicknesses ranging from 1 mm to 45 mm at a fixed diameter of 20 mm, as shown in Figure 2a–c. Experimental results are represented in Figure 2d–g. Figure 2a shows the representative modeling result for the normalized hydrogen desorption content versus the elapsed time for a diffusivity of 1 × 10^−10^ m^2^/s and five different thicknesses at a fixed diameter of 20 mm. The time-dependent normalized emission contents at the four different diffusivities were similar to those in Figure 2a. The normalized content was calculated as the emission content divided by the total uptake, [C(t)/C_∞_]. The black rectangle marked by blue arrows a, b, c, d and e in Figure 2a indicate the equilibrium times modeled at thicknesses of 1 mm, 3 mm, 7 mm, 15 mm and 45 mm, respectively. The equilibrium time obtained indicated the crossing point (black rectangle) on the x-axis between the normalized emission content line and 97% dotted line of the total sorption content shown with horizontal blue dotted line. Figure 2b depicts the desorption equilibrium time versus the squared thickness for five different diffusivities at a fixed diameter of 20 mm. The results were obtained using the numerical modeling and an exponential growth relationship was observed between the equilibrium time and the squared thickness with good squared correlation coefficients (R^2^ = 0.99), as predicted by Equation (6).

Figure 2c displays the reciprocal equilibrium time versus the diffusivity for five different thicknesses at a fixed diameter of 20 mm. The results revealed a distinct linear relationship between the reciprocal equilibrium time and the diffusivity with perfect squared correlation coefficients (R^2^ = 1.00), as predicted by Equation (5). Figure 2d,e displays the experimental results for desorption equilibrium time versus the squared thickness at a diameter of 12 mm for NBR and 9.6 mm for EPDM, respectively. The exponential growth behaviors in the experiment were consistent with the modeling result in Figure 2b. Figure 2f represents the experimental investigations revealing the linearity between the reciprocal equilibrium time versus diffusivity for a fixed diameter of 13.6 mm with a thickness of 2.5 mm, in which even though three points with low diffusivity are applied to Ar gas. Figure 2g also represents the experimental results showing the linearity between the reciprocal equilibrium time versus the diffusivity including different testing gases (N_2_ and O_2_). It means the linearity is valid regardless of species of testing gas. The gas independent linear dependence in Figure 2f,g supports the modeling results in Figure 2c.

**Figure 3:**
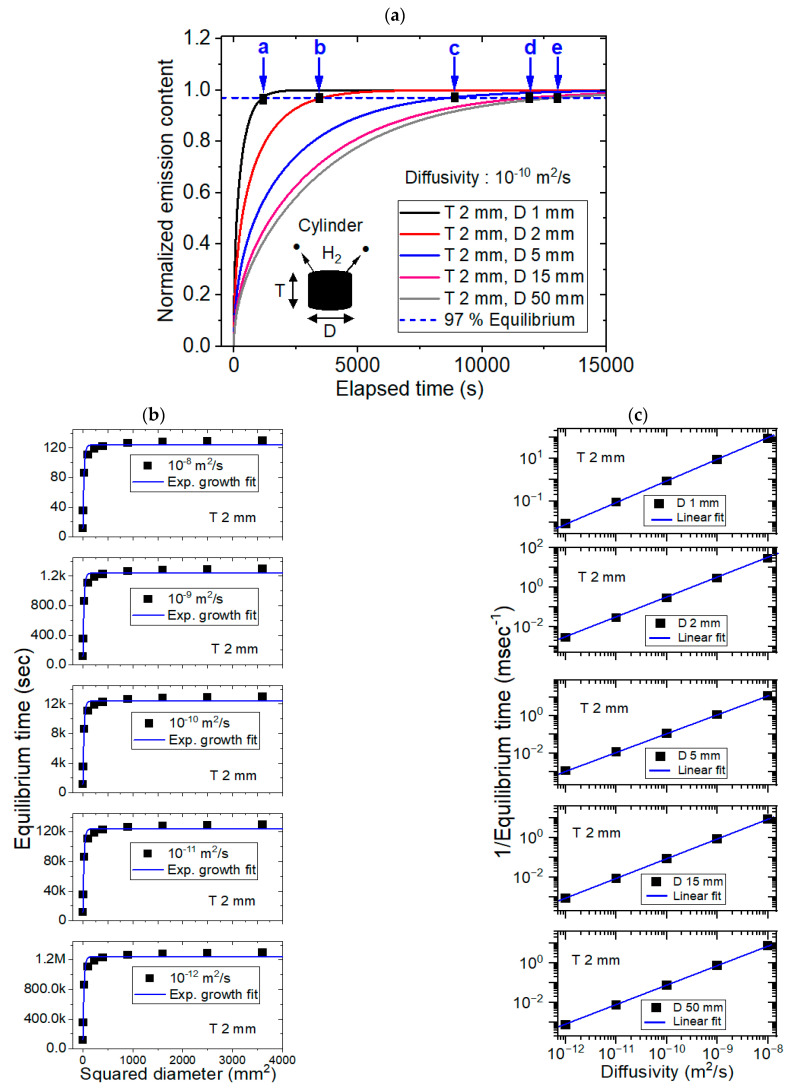

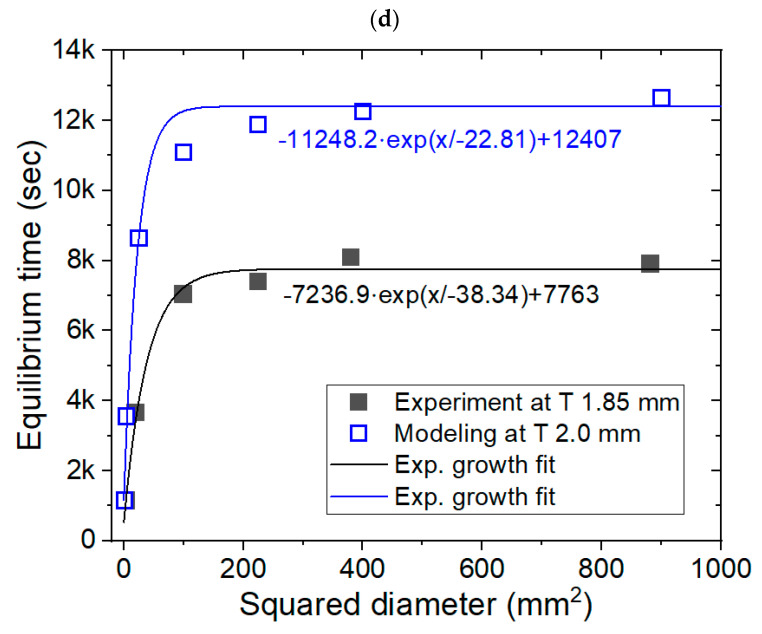
Modeling (**a**–**d**) and experimental (**d**) results for the cylinder-shaped specimen at a fixed thickness. (**a**) Numerical modeling for the normalized desorption hydrogen content versus the elapsed time for a diffusivity of 1 × 10^−10^ m^2^/s with five different diameters at a thickness of 2 mm. (**b**) Numerical modeling results for the hydrogen equilibrium time versus the squared diameter for five different diffusivities at a fixed thickness of 2 mm. (**c**) Linear correlation between the reciprocal equilibrium time and the diffusivity with five different diameters at a thickness of 2 mm. (**d**) Comparison between experiment and modeling. D and T in Figure 3 indicate the diameter and thickness, respectively, of the cylindrical specimen.

On the other hand, Figure 3 shows the modeling results for the cylinder-shaped specimen at a fixed thickness and experimental results. The modeling of cylinder-shaped specimens was also performed using a dedicated diffusion analysis program with five different diffusivities of 1 × 10^−8^ m^2^/s, 1 × 10^−9^ m^2^/s, 1 × 10^−10^ m^2^/s, 1 × 10^−11^ m^2^/s and 1 × 10^−12^ m^2^/s and diameters ranging from 1 mm to 60 mm at a fixed thickness of 2 mm. Figure 3a shows the representative modeling result for the normalized hydrogen desorption content versus the elapsed time for a diffusivity of 1 × 10^−10^ m^2^/s and five different diameters at a fixed thickness of 2 mm. The black rectangle marked by blue arrows, a, b, c, d and e in Figure 3a indicate the equilibrium times of 97% of the total sorption content modeled at diameters of 1 mm, 2 mm, 5 mm, 15 mm and 50 mm, respectively. The equilibrium time obtained indicated the crossing point (black rectangle) on the x-axis between the normalized emission content line and the 97% dotted line of the total sorption content shown by the horizontal blue dotted line. The time-dependent normalized emission contents at the four different diffusivities were similar to those in Figure 3a.

Figure 3b depicts the desorption equilibrium time versus the squared diameter for five different diffusivities at a thickness of 2 mm. The results showed an exponential growth relationship between the squared diameter and the equilibrium time with good squared correlation coefficients (R^2^ = 0.98), as predicted by Equation (7). Figure 3c displays the reciprocal equilibrium time versus the diffusivity for five different diameters at a fixed thickness of 2 mm. The results revealed a linear relationship between the reciprocal equilibrium time and the diffusivity with perfect squared correlation coefficients (R^2^ = 1.00), as predicted by Equation (5). The overall behaviors with different diffusivities, diameters and thicknesses were similar to those in Figure 3. Figure 3d shows the comparison between experiment and modeling, which reveals same exponential growth behavior between the desorption equilibrium time versus the squared diameter. In Figure 3d, the difference in saturation value of equilibrium time is attributed to different thicknesses. Therefore, the experimental result supports again the modeling results in Figure 3b.

### 3.2. Modeling for the Sphere-Shaped Polymer Specimens and Comparison with Experimental Results

**Figure 4:**
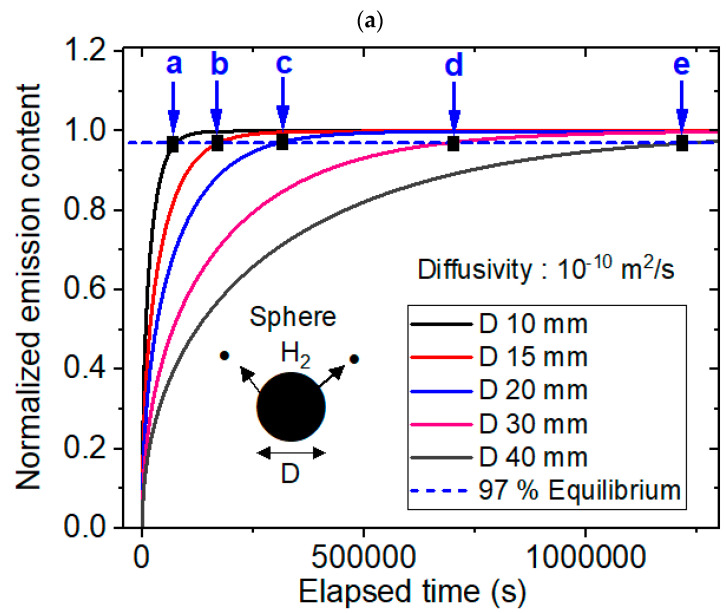

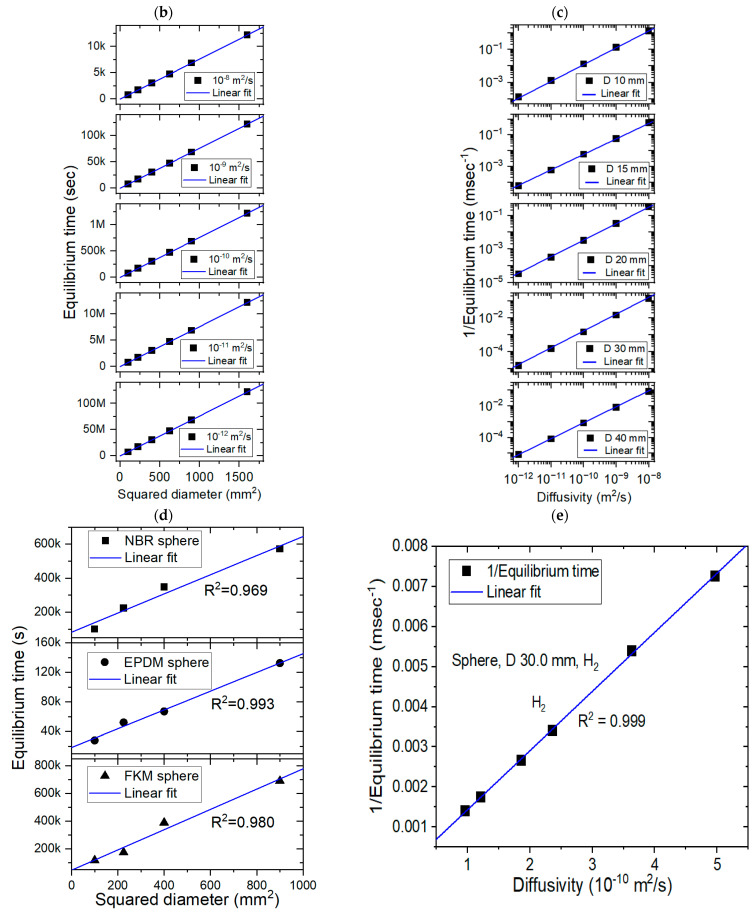

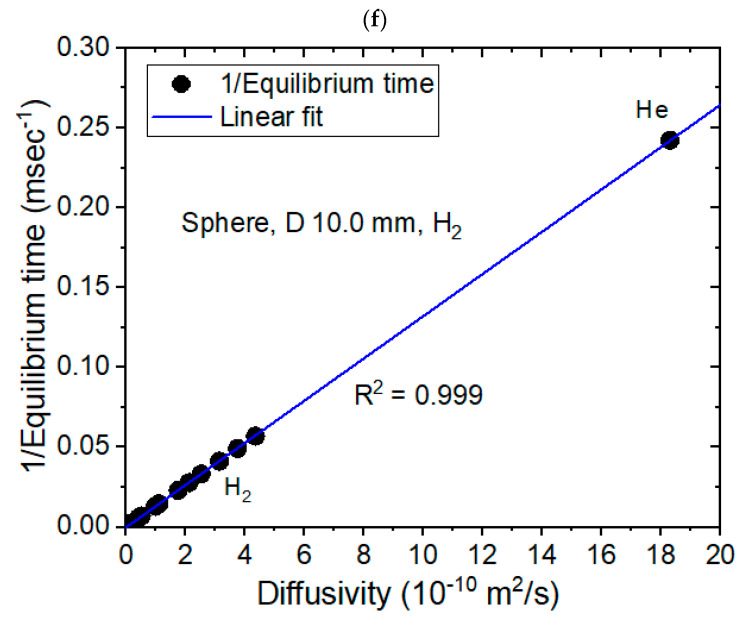
Modeling (**a**–**c**) and experimental (**d**–**f**) results for the sphere-shaped polymer. (**a**) Normalized hydrogen desorption content versus the elapsed time for a fixed diffusivity of 1 × 10^−10^ m^2^/s and five different diameters. (**b**) Linear correlation between the sorption equilibrium time and the squared diameter at five different diffusivities. (**c**) Linear correlation between the reciprocal equilibrium time and the diffusivity with five different diameters. (**d**) Experimental investigations with the desorption equilibrium time versus the squared diameter for three samples. (**e**) Experimental results with linearity between reciprocal equilibrium time versus the diffusivity. (**f**) Experimental results including He gas with linearity between reciprocal equilibrium time versus the diffusivity. D in Figure 4 indicates the diameter of the spherical specimen.

Figure 4 shows the modeling results for the spherical specimens, together with experimental results. Modeling of sphere-shaped polymer specimens was also performed using a dedicated diffusion analysis program with five different diffusivities of 1 × 10^−8^ m^2^/s, 1 × 10^−9^ m^2^/s, 1 × 10^−10^ m^2^/s, 1 × 10^−11^ m^2^/s and 1 × 10^−12^ m^2^/s and diameters ranging from 10 mm to 40 mm. Figure 4a shows the representative modeling result for the normalized hydrogen desorption content versus the elapsed time for a fixed diffusivity of 1 × 10^−10^ m^2^/s with five different diameters. The black rectangle marked by blue arrows a, b, c, d and e in Figure 4a indicate the equilibrium times modeled at diameters of 10 mm, 15 mm, 20 mm, 30 mm and 40 mm, respectively. Figure 4b shows the desorption equilibrium time versus squared diameter; here, a linear relationship was observed for the squared diameter for five diffusivities with perfect squared correlation coefficients (R^2^ = 1), as predicted in the Section 2. Figure 4c displays the reciprocal equilibrium time versus diffusivity for the five different diameters. The results also showed a linear relationship between the reciprocal equilibrium time and the diffusivity with perfect squared correlation coefficients (R^2^ = 1.00), as predicted by Equation (9). The overall behaviors with different diffusivities were similar to those in Figure 4b,c. Figure 4d shows the experimental investigations with the linearity between the desorption equilibrium time versus the squared diameter for three polymers (NBR, EPDM and FKM). The experimental linear behaviors coincided with the modeling results in Figure 4b. Figure 4e,f represents the experimental results with linear relationship between the reciprocal equilibrium time versus the diffusivity. The linear dependency was similar to the modeling results in Figure 4c.

### 3.3. Modeling for the Sheet-Shaped Polymer Specimens and Comparison with Experimental Results

**Figure 5:**
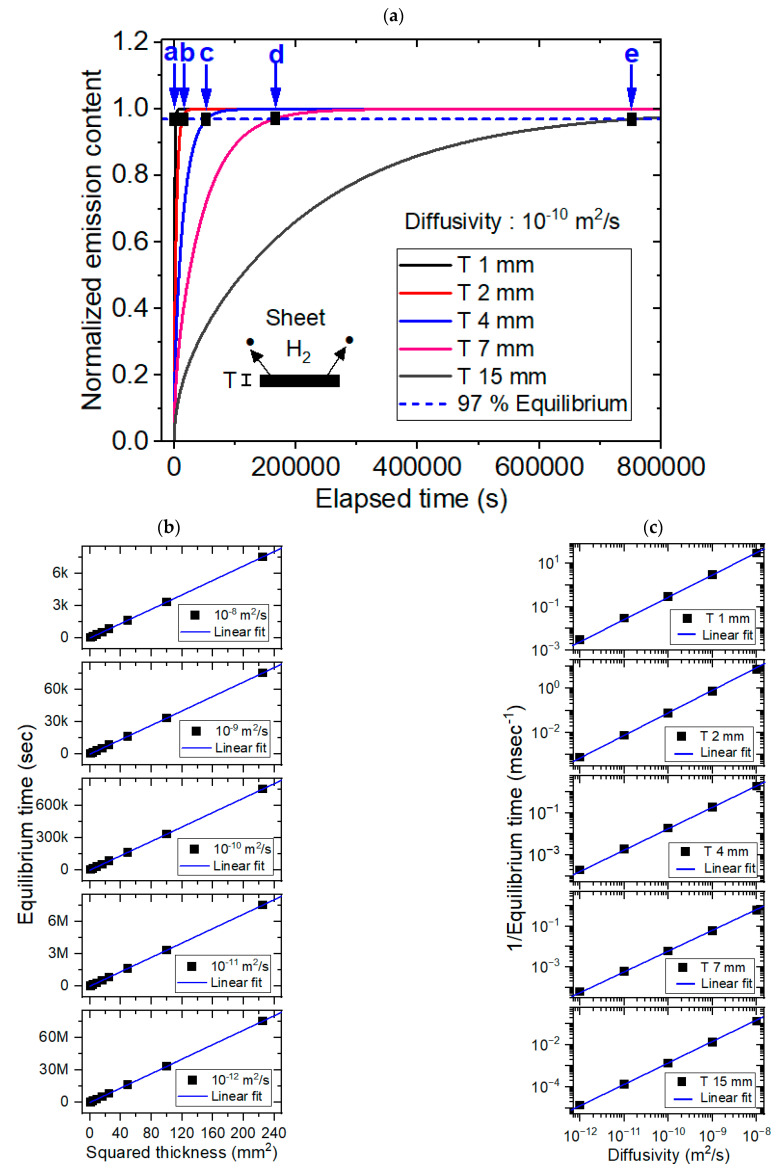

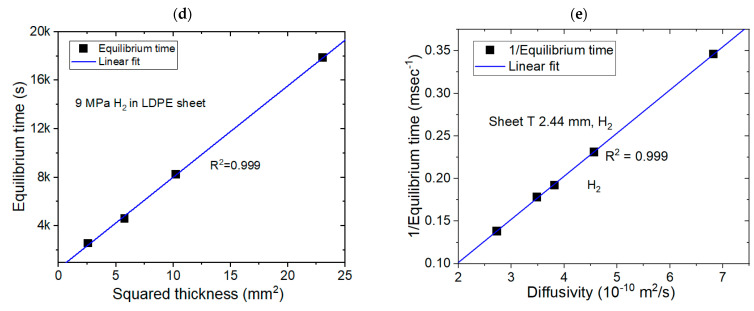
Modeling (**a**–**c**) and experimental (**d**,**e**) results for the sheet-shaped polymer. (**a**) Normalized hydrogen desorption content versus elapsed time for a fixed diffusivity of 1 × 10^−10^ m^2^/s and five different thicknesses. (**b**) Linear correlation between the sorption equilibrium time and the squared thickness for the five different diffusivities. (**c**) Linear correlation between the reciprocal equilibrium time and diffusivity with five different thicknesses. (**d**) Experimental investigations showing linearity between the desorption equilibrium time versus the squared thickness for LDPE. (**e**) Experimental results with the linearity between the reciprocal equilibrium time versus the diffusivity. T in Figure 5 indicates the thickness of the sheet specimen.

Figure 5 shows the modeling results for the sheet-shaped specimens, together with experimental results. Modeling of the sheet-shaped polymer specimens was also performed using a dedicated diffusion analysis program with five different diffusivities of 1 × 10^−8^ m^2^/s, 1 × 10^−9^ m^2^/s, 1 × 10^−10^ m^2^/s, 1 × 10^−11^ m^2^/s and 1 × 10^−12^ m^2^/s and thicknesses ranging from 1 mm to 15 mm. Figure 5a shows the representative modeling result for normalized hydrogen desorption content versus the elapsed time with a fixed diffusivity of 1 × 10^−10^ m^2^/s and five different thicknesses. The black rectangle marked by blue arrows a, b, c, d and e in Figure 5a indicate the equilibrium times modeled at thicknesses of 1 mm, 2 mm, 4 mm, 7 mm and 15 mm, respectively. Figure 5b shows the desorption equilibrium time; here, a linear relationship was observed with the squared thickness for the five different diffusivities with perfect squared correlation coefficients (R^2^ = 1), as predicted in the modeling background section. Figure 5c shows the reciprocal equilibrium time versus diffusivity for the five different thicknesses. The results revealed a linear relationship between reciprocal equilibrium time and diffusivity with perfect squared correlation coefficients (R^2^ = 1.00), as predicted in the Section 2. The overall behaviors with different diffusivities were similar to those in Figure 5b,c. Figure 5d shows the experimental investigations with linearity between the desorption equilibrium time versus the squared thickness for fixed diffusivities. The linearity behaviors in experiment were well similar to modeling results in Figure 5b. Figure 5e shows the experimental investigations with linearity between the reciprocal equilibrium time versus the diffusivity. The experimental linear behaviors were good agreement in the modeling results in Figure 5c.

As a similar diffusion analysis program, Yang et.al developed the program for calculation of diffusion coefficient based on the unipore model [26]. In order to automatically and time-effectively analyze the sorption–diffusion data, a matlab-based computer program was developed based on a least-squares criterion to regress the experimental gas sorption kinetic data and determine the corresponding diffusion coefficient. The developed program was limitedly applied to the sphere-shaped specimen according to Equation (9). Meanwhile, the developed diffusion analysis program in this work was applied to specimens with cylindrical, spherical and sheet shapes.

## 4. Conclusions

Modeling of the time-dependent H_2_ emission content using a dedicated diffusion analysis program was conducted for cylinder-, sphere- and sheet-shaped polymer specimens. The desorption equilibrium time needed to be determined before the periodic cyclic testing and the high-pressure H_2_ exposure. By utilizing the validated analysis program, we also modeled the H_2_ desorption equilibrium time with respect to the volume dimension in the cylinder-, sphere- and sheet-shaped polymer specimens with different diffusivities.

The equilibrium time for the desorption processes showed the exponential growth behavior with respect to the squared thickness and the squared diameter of the cylinder-shaped specimen; however, the equilibrium time was found to be linear to the squared diameter for the sphere-shaped specimen and the squared thickness for the sheet-shaped specimen. For all cylinder-, sphere- and sheet-shaped polymers investigated, a linear relationship was observed between the reciprocal equilibrium time and the diffusivity. In summary, the desorption equilibrium times were mainly affected by the following important factors: the diffusion coefficient, sample thickness and diameter.

The modeling results were confirmed by analysis of the solutions with respect to the complicated Fick’s second diffusion law. The modeling is in good agreement with experimental investigation results. The coincidence between modeling and experiment in gas diffusion are demonstrated at first time in the work. Thus, the modeling could predict the desorption equilibrium time for any shape of cylinder, sheet and sphere without measurement. Consequently, the time-dependent emitted H_2_ concentration behavior of multi-exponential form with a known diffusivity could be predicted for three shaped samples by applying of a diffusion analysis program.

## Figures and Tables

**Figure A1:**
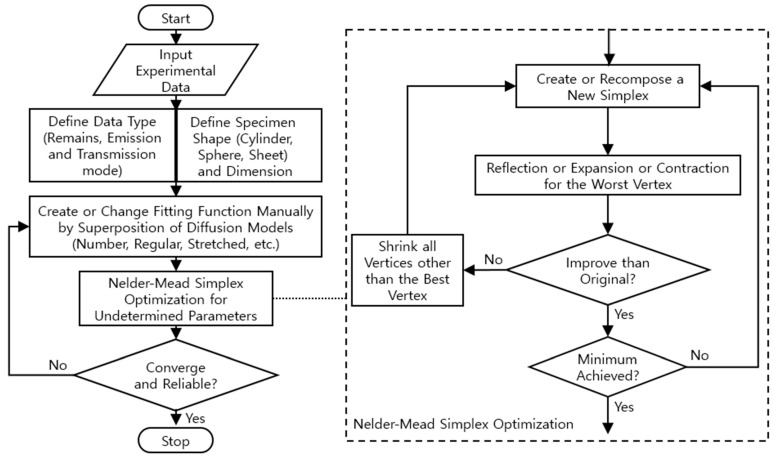
Flowchart for analyzing the gas uptake and diffusivity of specimens with different data types and various shapes with the Nelder–Mead simplex nonlinear optimization algorithm.

**Figure A2:**
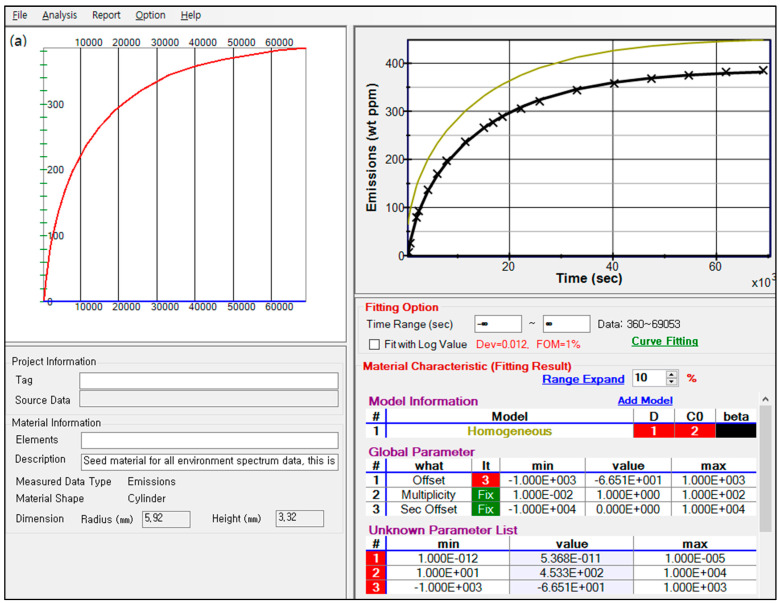
Application example of the diffusion analysis program to obtain the hydrogen uptake and diffusion coefficient for NBR H60 exposed to 8.9 MPa.

## Data Availability

The original contributions presented in the study are included in the article, further inquiries can be directed to the corresponding author.

## Appendix A. Flowchart, Application Example and Code for Diffusion Analysis Program

**<Coding of sheet, sphere- and cylinder-shaped specimen in the emission mode>**						
// Total amount of diffusing gas which has entered the sheet at time t						
// Ref: Crank et.al. The Mathematics of Diffusion (1975) p. 47, Equation (4.18) [24]						
// D: Diffusion coefficient, L: Sheet thickness						
// M: Emitted total amount by diffusion at infinite time.						
public double Ftn_Sheet(double t) {						
	double pi2 = Math.PI × Math.PI;					
	double zSum = 0;					
	for (int *n* = 0; *n* < 50; ++n) {					
		int n21 = (2 × *n* + 1) × (2 × *n* + 1);				
		double add = Math.Exp(−D × n21 × pi2 × t/(L × L))/n21;				
		double zSumAdded = zSum + add;				
		if (zSum >= zSumAdded) break; // When convergence achieved				
		zSum = zSumAdded;				
	}					
	return M − 8/pi2 × (M × zSum);					
}						

// Total amount of diffusing gas which has entered the sphere at time t.						
// Ref: Crank et.al. The Mathematics of Diffusion (1975) p. 91, Equation (6.20) [24]						
// D: Diffusion Coefficient, a: Radius of sphere.						
// M: Emitted total amount by diffusion at infinite time.						
public double Ftn_Sphere(double t) {						
	double pi2 = Math.PI × Math.PI;					
	double zSum = 0;					
	for (int *n* = 1; *n* < 100; ++n) {					
		int n2 = *n* × *n*;				
		double add = Math.Exp(−D × n2 × pi2 × t/(a × a))/n2;				
		double zSumAdded = zSum + add;				
		if (zSum >= zSumAdded) break; // When convergence achieved				
		zSum = zSumAdded;				
	}					
	return M − 6/pi2 × (M × zSum);					
}						

// Total amount of diffusing gas which has entered the cylinder at time t.						
// Ref: Al Demarez, et. al., Acta Metallurgica (1954), Equation (5) [28]						
// D: Diffusion Coefficient, a: Radius of cylinder, L: Length of cylinder						
// M: Emitted total amount by diffusion at infinite time.						
public double Ftn_Cylinder(double t) {						
	double pi2 = Math.PI × Math.PI;					
	double zSum = 0;					
	for (int *n* = 0; *n* < 100; ++n) {					
		int n2 = (2 × *n* + 1) × (2 × *n* + 1);				
		double add = Math.Exp(−n2 × pi2 × D × t/(L × L))/n2;				
		double zSumAdded = zSum + add;				
		if (zSum >= zSumAdded) break; // When convergence achieved				
		zSum = zSumAdded;				
	}					
	double rSum = 0;					
	for (int *n* = 1; *n* <= 50; ++n) {					
		double b = Bessel0root[*n* − 1];				
		double add = Math.Exp(−D × b × b × t/(a × a))/(b × b);				
		double rSumAdded = rSum + add;				
		if (rSum >= rSumAdded) break; // When convergence achieved				
		rSum = rSumAdded;				
	}					
	return M − 32/pi2 × (M × zSum × rSum);					
}						

// Zeros of first kind Bessel function J_0(x)						
public static double[] bessel0root =						
	new double[] {2.40482556, 5.52007811, 8.65372791, 11.79153444, 14.93091771,					
		18.07106397, 21.21163663, 24.35247153, 27.49347913, 30.63460647,				
		33.77582021, 36.91709835, 40.05842576, 43.19979171, 46.34118837,				
		49.4826099, 52.62405184, 55.76551076, 58.90698393, 62.04846919,				
		65.1899648, 68.33146933, 71.4729816, 74.61450064, 77.75602563,				
		80.89755587, 84.03909078, 87.18062984, 90.32217264, 93.46371878,				
		96.605267951, 99.74681986, 102.88837425, 106.02993092, 109.17148965,				
		112.3130503, 115.45461265, 118.59617663, 121.73774209, 124.87930891,				
		128.020877, 131.1624463, 134.3040166, 137.445588, 140.5871604,				
		143.7287336, 146.8703076, 150.0118825, 153.153458, 156.2950343};				

// Nelder-Mead Simplex Optimization.						
// Return value is fit error						
// Optima(class): Manage all optimization procedure and calc. error and FOM etc.						
// Simplex(Class): Polytope of N + 1 vertices in N dim. undetermined param. space.						
// Vertex(Class): One of N + 1 vertices of Simplex.						
// Coord(Class): Having coord. of vertex and methods, reflect(), expand(), …						
// simplexProtocol: ExpFactor = 2.0, ConFactor = 0.5, PrcntInitCoef = 5.0, …						
public double Iterate(double[] unknowns) {						
	int b, w;					
	int loop = 0; // # of loops					
	int dim = unknowns.Length;					
	Simplex simplex = new Simplex(unknowns, simplexProtocol.PrcntInitCoef);					
	// Calculate errors for each vertexes.					
	for (int i = 0; i < dim + 1; ++i)					
	simplex.s[i].error = optima.ErrorF(simplex.s[i].coord);					
	do { // start iteration					
		b = simplex.bestVertex(); // find best and worst vertices				
		w = simplex.worstVertex();				
		// calculate the midPoint of the non-worst values for later calc’s				
		double[] mp = simplex.midPoint(w);				
		Vertex refl_vrtx = new Vertex(simplex.s[w]); // save the old values				
		Coord.reflect(mp, refl_vrtx.coord); // first try a reflection				
		refl_vrtx.error = optima.ErrorF(refl_vrtx.coord);				

		// is the new vertex better or equal to the old best vertex				
		if (refl_vrtx.error < simplex.s[b].error) {				
			// try to expand the reflected vertex next			
			Vertex expd_vrtx = new Vertex(refl_vrtx);			
			Coord.expand(mp, expd_vrtx.coord, simplexProtocol.ExpFactor);			
			expd_vrtx.error = optima.ErrorF(expd_vrtx.coord);			
			// if the expanded vertex is not as good or better than the best			
			// revert to the reflected vertex			
			if (expd_vrtx.error <= refl_vrtx.error) simplex.s[w] = expd_vrtx;			
			else simplex.s[w] = refl_vrtx;			
		} else {				
			if (refl_vrtx.error <= simplex.s[w].error) simplex.s[w] = refl_vrtx;			
			else {			
				Vertex cont_vrtx = new Vertex(simplex.s[w]);		
				Coord.contract(mp, cont_vrtx.coord, simplexProtocol.ConFactor);		
				cont_vrtx.error = optima.ErrorF(cont_vrtx.coord);		
				if (cont_vrtx.error <= simplex.s[w].error) simplex.s[w] = cont_vrtx;		
				else {		
					simplex.contract_all(b, simplexProtocol.ConFactor);	
					for (int i = 0; i < dim + 1; ++i)	
						simplex.s[i].error = otima.ErrorF(simplex.s[i].coord);
				}; // else		
			}; // else			
		};				
		++loop;				
	} while (!(simplex.s[b].error <= simplexProtocol.Tolerance ||					
	loop >= simplexProtocol.InternalLoop));					
	// return best result					
	for (int i = 0; i < dim; ++i) unknowns[i] = simplex.s[b].coord[i];					
	return simplex.s[b].error;					
}						
